# PTSD symptoms and coping before and during COVID-19 pandemic among help-seeking veterans: prospective cohort study

**DOI:** 10.1192/j.eurpsy.2022.215

**Published:** 2022-09-01

**Authors:** M. Letica-Crepulja, A. Stevanović, D. Palaić, T. Grahovac Juretić, J. Grković, I. Rončević-Gržeta

**Affiliations:** 1 Clinical Hospital Center Rijeka, Department Of Psychiatry, Rijeka, Croatia; 2 Faculty of Medicine, University of Rijeka, Department Of Psychiatry And Psychological Medicine, Rijeka, Croatia; 3 Faculty of Health Studies University of Rijeka, Department Of Basic Medical Sciences, Rijeka, Croatia

**Keywords:** posttraumatic stress disorder, Covid-19, Veterans

## Abstract

**Introduction:**

The COVID-19 pandemic has threatened the mental health of individuals around the world. Ex-combatants have been repeatedly shown to be increased risk of experiencing social and psychological problems during emergencies.

**Objectives:**

To compare the severity of overall posttraumatic stress disorder (PTSD) symptoms and PTSD clusters among help-seeking veterans before and during the COVID-19 pandemic. The second aim was to identify coping strategies used and track possible changes during the timeline.

**Methods:**

Male war veterans receiving outpatient treatment at the Referral Center for PTSD were assessed at baseline (t1=12-18 months before the COVID-19 pandemic), during the first lockdown (March-June 2020) and 12 months after the beginning of the COVID-19 pandemic (March-June 2021). A total of 132 veterans participated in all three measurements. The Life Events Checklist for DSM-5 (LEC-5), PTSD Checklist for DSM-5 (PCL-5), and The Brief COPE were used.

**Results:**

Exposure to COVID-19 pandemic related stressors increased over time. The great majority of participants (91.0%) followed the preventive measures. The severity of the overall PTSD symptoms significantly decreased during timeline (t1=56.9, 11.15; t2= 47.24, SD=12.87; t3= 44.1, SD=14.09). At t2, all participants still fulfilled the PTSD diagnostic criteria, and at t3, 23 participants (17.42%) did not meet all of the criteria for PTSD. The participants used adaptive coping rather than dysfunctional coping during the pandemic.

**Conclusions:**

Despite the expectations of worsening the symptoms, help-seeking veterans with PTSD appeared to develop adaptive adjustment to the COVID-19 pandemic stressors, which is in line with the results of the recent longitudinal research and will be discussed.

**Disclosure:**

No significant relationships.

